# *Bacillus cytotoxicus* Isolated from a Pristine Natural Geothermal Area Reveals High Keratinolytic Activity

**DOI:** 10.3390/microorganisms8060796

**Published:** 2020-05-26

**Authors:** Ivana Cavello, María Sofía Urbieta, Sebastián Cavalitto, Edgardo Donati

**Affiliations:** CINDEFI (CCT La Plata-CONICET, UNLP), Facultad de Ciencias Exactas, Universidad Nacional de La Plata, Calle 47 y 115, (B1900ASH) La Plata 1900, Argentina; ivanacavello@gmail.com (I.C.); cavalitto@quimica.unlp.edu.ar (S.C.); donati@quimica.unlp.edu.ar (E.D.)

**Keywords:** Domuyo geothermal area, keratinases, feather waste, cytK-1 toxin

## Abstract

Geothermal areas are the niches of a rich microbial diversity that is not only part of the intangible patrimony of a country but also the source of many microbial species with potential biotechnological applications. Particularly, microbial species in geothermal areas in Argentina have been scarcely explored regarding their possible biotechnological uses. The purpose of this work was to explore the proteolytic and keratinolytic enzymatic potential of microorganisms that inhabit in the Domuyo geothermal area in the Neuquén Province. To this end, we did enrichment cultures from two high-temperature natural samples in mineral media only supplemented with whole chicken feathers. After the isolation and the phylogenetic and morphologic characterization of different colonies, we obtained a collection of *Bacillus cytotoxicus* isolates, a species with no previous report of keratinolytic activity and only reported in rehydrated meals connected with food poisoning outbreaks. Its natural habitat has been unknown up to now. We characterized the proteolytic and keratinolytic capacities of the *B.* *cytotoxicus* isolates in different conditions, which proved to be remarkably high compared with those of other similar species. Thus, our work represents the first report of the isolation as well as the keratinolytic capacity characterization of strains of *B. cytotixicus* obtained from a natural environment.

## 1. Introduction

Nowadays, it is fairly known that geothermal environments are the niches of a rich prokaryotic biodiversity, formed by extremophilic, in certain cases novel microorganisms, which can present high biotechnological potential in many fields [1]. Patagonia in the southwest of Argentina presents two volcanic geothermal areas—Copahue and Domuyo. The Copahue geothermal area, predominantly acidic, has been extensively studied as for its prokaryotic biodiversity [2,3,4] and the biotechnological applications of some of their species [5,6,7]. On the other hand, the Domuyo geothermal area remains almost unexplored in both aspects. The Domuyo geothermal area is located on the northwest of the Neuquén Province, Argentina, about 550 km of the city of Neuquén, the provincial capital. It is on the southwestern slope of Cerro Domuyo (36°340 S, 70°250 W), the highest mountain in Patagonia with an elevation of 4709 meters above sea level. The geothermal area presents many surface manifestations, including fumaroles, hot springs, and geysers with neutral pH [8].

In the field of biotechnological potential of environmental microorganisms, thermophiles, either moderate or hyper, have gain massive attention in the last few years, especially because of the remarkable properties of their enzymes [1,9]. Among such extremozymes, hydrolytic enzymes able to catalyze the degradation of waste materials are of enormous importance for certain industries, since they can aid in the conversion of massive volumes of waste into profitable products. A notorious example is the use of keratinases by the poultry industry. The poultry industry is continuously expanding to attend the increasing worldwide demand for meat and other poultry products. Such an increase inevitably generates high amounts of organic wastes like feathers and viscera [10]. Feathers are mainly composed by keratins, which are structural proteins packed into a supercoiled polypeptide chain extensively cross-linked with disulphide bridges, hydrogen bonds, and hydrophobic interactions [11]. Despite their insoluble and recalcitrant structure, keratins can be efficiently hydrolyzed by keratinolytic enzymes produced by many bacteria and fungi [11,12]. In particular, the bacterial genus *Bacillus* is among the most reported producer of keratinolytic proteases [13,14,15]. A fairly large number of mesophilic keratinolytic microorganisms have been studied [16,17], and some of them can even produce keratinases that are active at higher-temperature conditions [18,19]. In addition, many proteases have been discovered in thermophilic and hyperthermophilic bacteria [20,21,22]. However, only few thermophilic bacteria are known to produce keratinases. Thus, there is still a need to find more robust and specific thermostable keratinolytic enzymes, which could be applied to fulfill the increasing demand of detergent, leather, textile, food, and pharmaceutical industries [12,22,23,24,25,26,27,28].

In such context, we aimed for the isolation and characterization, with emphasis on their proteolytic and keratinolytic capacity, of microorganisms with the ability to degrade chicken feathers from the little explored geothermal area of Domuyo (the Neuquén Province in Argentina).

## 2. Materials and Methods

### 2.1. Samples Collection

Samples consisting of a mixture of water and biofilms were collected from the manifestation Las Olletas (abbreviated as “Oll” to designate isolates obtained from these samples) and Los Tachos (abbreviated as “LT”) in the Domuyo geothermal area. In both places, the samples consisted in equal parts of water and biofilms manually collected in sterile plastic tubes with a volume of 50 mL and kept at room temperature. Both environmental samples had temperatures between 50 °C and 60 °C and a pH value of 7.0. Las Olletas is located on top of the valley of the Manchana Covunco. Meanwhile, Los Tachos is located on the valley of the Cavunco stream, where thermal manifestations are dominated by high-temperature waters and high concentrations of sodium chloride [29].

Temperature and pH were measured in situ with a Hanna HI 8424 portable instrument (Hanna Instruments, Woonsocket, RI, USA) properly calibrated against calibration standards.

### 2.2. Isolation of Keratinolytic Microorganisms

Approximately 1 g of samples collected from Los Tachos or Las Olletas were inoculated in 500 mL Erlenmeyer flasks containing 50 mL of mineral media (MM; described below) supplemented with whole feathers as sole carbon and nitrogen sources. Cultures were incubated at 50 °C in an orbital shaker at 150 rpm until the visual confirmation of feather degradation in the liquid medium (1 to 5 days). For the isolation of keratinolytic microorganisms, 100 µL of each enrichment culture and 100 µL of 1/10 and 1/100 dilutions were spread onto a nutrient broth (NB) medium solidified with 2% *w*/*v* agar and incubated at 50 °C. Bacterial isolates were grouped according to the sample of origin and the macroscopic characteristics of colonies, such as pigmentation, texture, elevation, and size. Single colonies were streaked twice on NB agar plates to obtain microbial monocultures.

The formulation of the MM was: NaH_2_PO_4_, 0.496 g·L^−1^; K_2_HPO_4_, 2.486 g·L^−1^, and 0.50 mL of a trace elements solution (FeCl_3_·6H_2_O, 0.016 g·L^−1^; ZnCl_2_, 0.013, g·L^−1^; MgCl_2_, 0.010 g·L^−1^; and CaCl_2_, 0.00011 g·L^−1^) adjusted at pH 7.0 [30]. The MM were supplemented with 10 g·L^−1^ of chicken feathers. Chicken feathers were supplied by a local slaughterhouse, washed thoroughly with 1g·L^−1^ sodium dodecyl sulphate (SDS) to remove surface contaminants and then washed with a 1:1 (*v*/*v*) methanol:water mixture by shaking at 28 °C for 18 h; afterwards, they were rinsed with distilled water and then with 95% ethanol and were finally air-dried.

### 2.3. Proteolytic Potential on Solid Media

The isolated bacteria were initially screened for extracellular protease production in skim milk agar plates. After incubation at 50 °C for 24–48 h, the plates were observed for the presence of clear zones around the colonies, which indicated the production of proteolytic enzymes. Clearance zone (H) and colony diameter (C) were measured, and the H/C ratio was calculated.

Bacteria displaying protease production in skim milk agar were inoculated into feather meal agar (FMA) plates to search for keratinolytic activity. After inoculation, the plates were incubated at 50 °C for 48 h, and bacterial growth as well as the presence of hydrolysis halo around the colonies was checked visually [31].

### 2.4. Qualitative Evaluation of Feather Degradation

The isolates that showed proteolytic activity were evaluated for their feather-degrading potential on test tubes containing 10 mL of MM and a single chicken feather. Assays were performed in duplicate. The tubes were incubated at 50 °C and centrifuged at 150 rpm for up to 5 days. The tubes were checked on a daily basis, and the isolates that showed keratinolytic activity (feather degradation) were selected for the subsequent taxonomic identification and characterization of their extracellular enzymatic activities and the capability of growth at different temperatures, pH, and salt concentrations.

### 2.5. Identification of Feather-Degrading Isolates: DNA Extraction, 16S rRNA Gene Amplification, and Cytotoxin K-1 (CytK-1) Gene Amplification

The genomic DNA of each feather-degrading isolate was extracted as described by Park [32]. The 16S rRNA gene of each isolate was amplified using the primer set 27F: 5′-AGAGTTTGATCCTGGCTCAG 3′ and 1541R: 5′-AAGGAGGTGATCCAGCCGCA-3′. PCR was done according to Urbieta et al. [3]. PCR amplification was checked by 1.5% agarose gel electrophoresis stained with ethidium bromide. The 16S rRNA gene product of each isolate was sequenced using Macrogen services (Macrogen Inc, Seul, Korea). The respective sequences were analyzed and compared with the NCBI database using BLASTn software (http://ncbi.nlm.nih.gov/BLAST). The phylogenetic analyses of the 16S rRNA gene of the isolates was done using MEGA version 6. The 16S rRNA gene sequences were aligned with the sequences of the homologous regions of closely related strains retrieved from the NCBI GenBank. Evolutionary distances were computed using the Kimura two-parameter model, and a phylogenetic tree was obtained by the maximum likelihood method [33]. All positions containing alignment gaps and missing data were eliminated in pairwise sequence comparisons (pairwise deletion option). The stability of clades was assessed with 1000 bootstrap replications. The 16S rRNA gene sequences of the isolates were deposited in the NCBI database, and the accession numbers are shown in the text.

The amplification of *cytK-1* gene was performed using primers CK1F and CK1R according to Guinebretiére et al. [34].

### 2.6. Biochemical and Extracellular Enzymatic Characterization of Bacillus Cytotoxicus (B. cytotoxicus) Isolates

*B. cytotoxicus* isolates were characterized by their capability of growth at different temperatures and different salt concentrations as well as their extracellular enzymatic profiles.

Growths at different temperatures (20–65 °C) were tested on NB media and monitored by measuring the growth media turbidity by optical density at 600 nm (OD_600nm_). Growths at different salt concentrations (NaCl: 0, 25, 50, 75, and 100 g·L^−1^) were tested on NB media at 50 °C and monitored by turbidity at OD_600nm_.

The biochemical characterization of *B. cytotoxicus* strains isolated from the Domuyo geothermal area in the Neuquén Province was done by standard biochemical tests such as the determination of presence of the enzymes catalase, cytochrome oxidase, nitrate reductase, tiosulphate reductase and cysteine desulphurilase, the consumption of glucose, lactose, mannitol, and citrate, the methy red test and the Voges–Proskauer test. The biochemical tests were performed using commercial media provided by Britania (Britania, Buenos Aires, Argentina).

Different culture-dependent detection assays were conducted on agar plates to evaluate the extracellular hydrolytic enzymatic profiles of the *B. cytotoxicus* isolates. The isolates were inoculated on the surface of the plates containing different media. The plates were incubated at 50 °C and examined after 24–48 h to detect the hydrolytic activities described below. A noninoculated plate served as a control.

The ability to degrade starch (amylolytic activity) was evaluated using starch agar, which consisted of 10 g·L^−1^ soluble starch (Mallinckrodt Baker Inc.), 2 g·L^−1^ yeast extract, 5 g·L^−1^ peptone, 0.5 g·L^−1^ MgSO_4_, 0.5 g·L^−1^ NaCl, 0.15 g·L^−1^ CaCl_2_, and 20 g·L^−1^ agar (adjusted at pH 6.0).

To detect pectinases, a selective medium containing 10 g·L^−1^ citric pectin (Sigma-Aldrich), 1.4 g·L^−1^ NH_4_SO_4_, 2.0 g·L^−1^ K_2_HPO_4_, 0.2 g·L^−1^ MgSO_4_·7H_2_O, 1 mL g·L^−1^ solution A (5 mg·L^−1^ FeSO_4_·H_2_O, 1.6 mg·L^−1^ MnSO_4_·H_2_0, and 2 mg·L^−1^ CoCl_2_) and 20 g·L^−1^ agar.

The production of extracellular inulinases was detected using inulin agar media, which consisted of 10 g·L^−1^ inulin, 2 g·L^−1^ yeast extract, 5 g·L^−1^ peptone, 0.5 g·L^−1^ MgSO_4_, 0.5 g·L^−1^ NaCl, 0.15 g·L^−1^ CaCl_2_, and 20 g·L^−1^ agar (adjusted to pH 6.0).

The production of amylases, pectinases, or inulinases was detected by the presence of a clear halo around the colonies after flooding the solid media plates with Lugol′s iodine solution [35].

Screening for cellulase activity was done on a carboxymethylcellulose (CMC) agar medium containing 2.0 g·L^−1^ NaNO_3_, 1.0 g·L^−1^ K_2_HPO_4_, 0.5 g·L^−1^ MgSO_4_, 0.5 KCl, 2.0 g·L^−1^ CMC sodium salt (Mallinckrodt Baker Inc.), 0.2 g·L^−1^ peptone, and 17 g·L^−1^ agar.

To detect xylanases, a xylan agar medium containing 10 g·L^−1^ xylan from birchwood (Sigma-Aldrich), 5.0 g·L^−1^ yeast extract, 5 g·L^−1^ peptone, 1 g·L^−1^ K_2_HPO_4_, 0.2 g·L^−1^ MgSO_4_·7H_2_O, and 20 g·L^−1^ agar.

The production of cellulases and/or xylanases was detected by the presence of a clear halo around the colonies after flooding the solid media plates with Congo Red solution (1 g·L^−1^) and destained with 1M NaCl [36].

### 2.7. Keratinase Production and Analytical Determination of Proteolitical Activity

The production of keratinases of the isolates LT-1, Oll-15, and Oll-16 was carried out in 250 mL Erlenmeyer flasks with 50 mL of MM supplemented with 10 g·L^−1^ of chicken feathers. Cultures were performed on a rotary shaker (180 rpm) at 50 °C, and the samples were taken periodically. The cultures supernatants were obtained by centrifugation at 10,000× *g* for 10 min and used as crude extracts for the analytic determination of the protease production.

Proteolytic activity was assessed using azocasein as a substrate. Briefly, 100 µL of properly diluted crude extract were added to 250 µL of 10 g·L^−1^ azocasein solution (Tris-HCl buffer: 20 mmol·L^−1^; pH: 7.5). After incubation at 50 °C for 30 min, the reaction was stopped by adding 1000 µL of trichloroacetic acid (TCA; 10%, *w*/*v*). The admixture was centrifuged at 10,000× *g* for 5 min, and 500 µL of the supernatant were added to 500 µL of NaOH (1.0 mol·L^−1^). Absorbance was measured at 440 nm. The assays were performed in triplicates, and suitable controls were prepared by adding TCA to the reaction mixtures before adding the crude extract. One unit (U) of protease activity was arbitrarily defined as the increase in 0.01 absorbance unit under the experimental conditions used.

All determinations were performed in three independent replicates. The experimental results were expressed as the mean of the replicate determinations and the standard deviation (mean ± SD).

## 3. Results

### 3.1. Molecular Identification of the Bacterial Isolates

**Figure 1:**
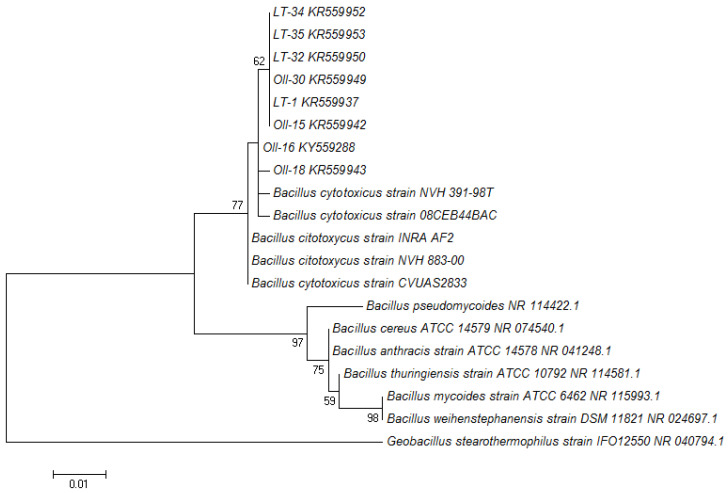
Phylogenetic tree of the 16S rRNA gene of the *Bacillus cytotoxicus* (*B. cytotoxicus*) isolated from Domuyo obtained by maximum likelihood (Kimura two-parameter (K2P) distance method). Bootstrap values (1000 tree interactions, shown in the unit of %) higher than 50 are indicated at the nodes. The scale bar represents 1% estimated phylogenetic diversity.

**Figure 2:**
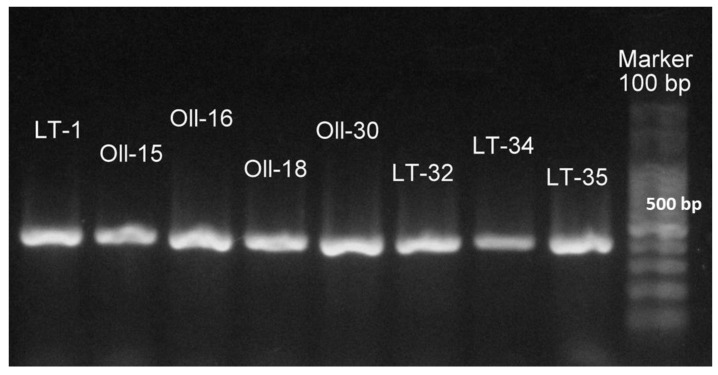
Gel image of the amplification of cytotoxin K-1 gene (426 bp) in the *B. cytotoxicus* isolated from Domuyo.

The 13 keratinolytic isolates obtained as described in Materials and Methods were identified through the 16S rRNA gene sequencing and the BLAST comparison. All of them were identified as members of the genus *Bacillus*. Five of the isolates shared 99% of sequence similarity with *Bacillus licheniformis* strain ATCC 14580, while eight isolates (named LT-1, Oll-15, Oll-16, Oll-18, Oll-30, LT-32, LT-34, and LT-35) shared more than 98% of sequence similarity with *B. cytotoxicus* NVH 391-98. The finding of these strains was particularly interesting, because all the previously isolated *B*. *cytotoxicus* reported so far have been found in samples related to food and food poison outbreaks [37,38]. To confirm the taxonomic affiliation of the *B. cytotoxicus* obtained from Domuyo, we constructed a phylogenetic tree (Figure 1), which showed that these isolates and their close relatives (*B. cytotoxicus* NVH 391-98^T^, INRA AF2, CVUAS2833, 08CEB44BAC, and NVH 883-00) formed a homogeneous and robust cluster easily differentiated from all the other *B. cereus* group species. In order to further confirm the phylogenetic classification, we amplified the gene that codifies for the *cytK-1* gene (426 bp), which has been used in a PCR diagnostic method to detect *B. citotoxicus* [34,37], with positive results for all the isolates affiliated with *B. cytotoxicus* (Figure 2).

### 3.2. Characterization of the Proteolytic and Keratinolytic Capacity of the Isolates

Table 1 shows the main characteristic of the 13 proteolytic isolates, including colony and hydrolysis halo diameters, taxonomic identification, and the accession number for the 16S rRNA sequences in the NCBI database. Ten of the isolates were able to produce hydrolysis halo on milk agar at 50 °C after 24 h, and three of them (LT-6, LT-7, and LT-8, shown in bold in Table 1) did it after 48 h. The H/C ratio is an indicator of the protease production efficiency. Thus, considering the data collected in Table 1, the isolates LT-1, Oll-15, and Oll-16 were the most efficient exhibiting the highest H/C ratio, and they were selected to study the time course production of their keratinolytic protease enzymes using a minimal mineral medium supplemented with whole chicken feathers (see below).

To further characterize the keratinolytic capacity of the isolates, they were tested on the FMA plates at 50 °C. All of the proteolytic isolates showed keratinolytic activity; 75% of them produced halo around the colonies after 48 h of cultivation, while the remaining 25% did it after 72 h. Finally, the isolates were evaluated for their ability to degrade whole chicken feathers, showing 58% of the isolates showed feather degradation after 48 h of incubation and 42% of them degraded the feathers completely after 72–96 h of incubation.

### 3.3. Quantification of the Keratinolitic Activity of the Most Efficient B. citotoxicus Isolates

**Figure 3:**
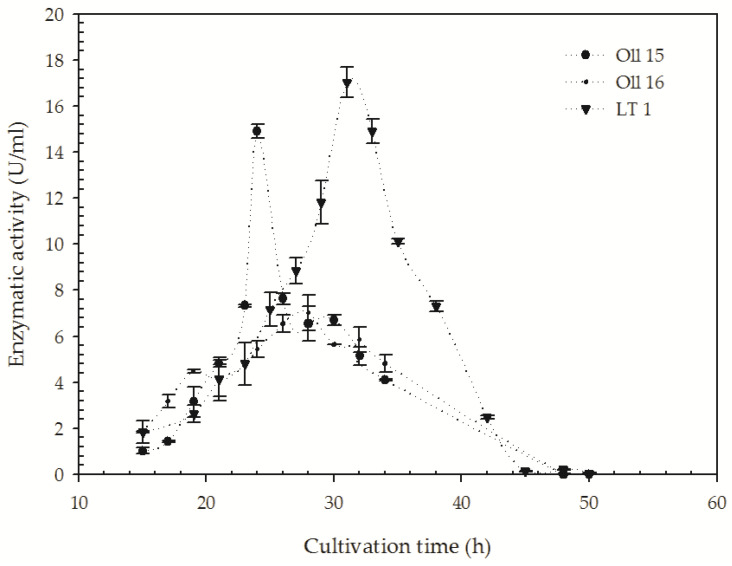
Keratinolytic protease production profiles of the *B. citotoxycus* strains. Strains were incubated in minimal media supplemented with 10 g·L^−1^ of whole feather at 50 °C for 50 h and centrifuged at 120 rpm.

It has been extensively reported for proteases with keratinolytic activity that both activities are linearly related consequently proteolytic activity, which can be used as an indirect measurement of keratinolytic activity [39]. Thus, protease activity was selected to follow up the time course production of keratinolytic activity in the isolates (LT-1, Oll-15, and Oll-16) that showed the highest H/C ratio (Table 1). As can be seen in Figure 3, proteolytic activity was successfully detected in cell-free supernatants. All the strains showed an increase in the proteolytic activity on azocasein over the first day with different cultivation times. For the isolate Oll-15, proteolytic activity reached the maximum in the first day (14.9 ± 0.3 U/mL), followed by that of Oll-16 (7.0 ± 0.6 U/mL), whereas the isolate LT-1 maximum protease activity was observed after 30 h of incubation (17.1 ± 0.4 U/mL). After achieving the maximum protease activity, decreases in their enzymatic titles were observed, which can be related to the stability of these enzymes in an alkaline environment with the culture. It has been reported that the activity or stability of keratinolytic proteases might be influenced by long incubation times on liquid media, probably due to the unfavorable impact on the enzyme production of the increasing pH value caused by the deamination reactions in the hydrolysis products of the keratinous substrates [39,40].

### 3.4. Biochemical and Extracellular Enzymatic Profile Characterization of the B. cytotoxicus Isolates

As part of the characterization of the first *B. cytotoxicus* strains isolated from a natural environment, we evaluated their ability to grow at different temperatures and salt concentrations as well as their extracellular enzymatic profiles.

The optimal growth for all the isolates occurred between 30 and 37 °C, while the minimum growth occurred at 20 and 50 °C and no growth was detected at 65 °C. It is worth mentioning that although the optimal growth for all the isolates was in the mesophilic range of temperatures, the degradation of whole chicken feathers was only observed at 50 °C. All the isolates were able to grow with 25 g·L^−1^ of NaCl, and more than 50% of them grew with 100 g·L^−1^ of NaCl. Thus, considering these results, the *B. cytotoxicus* strains isolated from Domuyo can be considered as moderate halophiles. These and other biochemical characteristics related with metabolic enzymes and pathways, the consumption of substrates and the production of gases and protons of the *B. cytotoxicus* strains are presented in Supplementary Table S1.

The hydrolytic enzymatic profiles of the isolates are presented in Supplementary Table S2. Briefly, all the *B. cytotoxicus* isolated showed keratinolytic activity, while three of them had pectinolytic activity (LT-1, Oll-15, and Oll-16) and only one had cellulase activity (Oll-15). None of the strains showed amylase, esterase, inulinase, or xylanase activities.

## 4. Discussion

Until today, the ability of thermophilic microorganisms to degrade feathers seems to be rare, and reports dealing with this subject are scarce. This scenario reveals the need to discover novel robust biocatalysts to solve the needs of poultry and other related industries. Thus, in an attempt to solve such a need, we assessed the keratinolytic potential of the microorganisms of the geothermal area of Domuyo.

Several keratinolytic thermophilic and termotolerant bacteria isolated from extreme environments have been reported up to now, including *Fervidobacterium pennivorans*, *F. islandicum* AW-1, *Thermoanaerobacter keratinophilus* 2KXI, *Caldanaerobacter* sp. strain 1523-1, and *Clostridium sporogenes bv. pennivorans*. However, all are anaerobic microorganisms, which hinder their practical utilization [12,22,23,24,25].

B. licheniformis PWD-1 [41], Streptomyces thermonitrificans MG104 [42], Meiothermus ruber H328 [26], Meiothermus sp. I40 [43], Bacillus subtilis (B. subtilis) RM-01 [44], Brevibacillus thermoruber T1E [45], B. halodurans JB99 [46], and Thermoactinomyces sp. YT06 [47] are representatives of keratinolytic bacteria that can be consider moderately thermophilic aerobic bacteria (50–60 °C). Among these, both Meiothermus species are the only ones obtained from hot spring environments, while all the others are isolated from samples of soil, waste streams, sugarcane molasses, or poultry wastes. In this sense, isolates from the Domuyo geothermal area are now enlarging the list of keratinolytic microorganisms isolates from hot springs.

The feather-degrading features of some aerobic, thermophilic bacteria reported in this work and in bibliography are summarized in Table 2. Compared with other keratinolytic bacteria, the *B. cytotoxicus* isolates from Domuyo presented in this work exhibited a higher feather consumption rate.

The keratinolytic activity of *B. licheniformis* strains has been extensively studied and well characterized [18,41,48,49,50], and even the keratinolytic enzyme Ker-A from the feather-degrading bacterium *B. licheniformis* PWD-1 was cloned and functionally expressed in *B. subtilis* [51]. On the other hand, there is no literature on the keratinolytic activity of *B. cytotoxicus*, and this work represents the first report on the subject.

Concerning to the production of keratinolytic protease by *B. cytotoxicus* species, it was observed that keratinases expression was inducible and occurred only in the presence of feathers as an exogenous inducer when it was incubated at 50 °C. Similar inducibility has been found in varieties of microorganisms [27,52,53].

For the three isolates, LT-1, Oll-15, and Oll-16, keratinolytic protease activity reached their maximum between 24 and 30 h of cultivation. These results are in concordance with some mesophilic microorganisms. For example, we can mention keratinase production by *Chryseobacterium* sp. RBT, where 30 h was found as an optimum incubation period for the maximum production [54], and the keratinase production by *B. cereus* TS1 with an optimum incubation time of 72 h [55]. An incubation period of two days was found for an optimum keratinase production by *Stenotrophomonas maltophila* R-13 [56] and *B. cereus* LAU08 [57]. Meanwhile, enzyme productions by *B. pumilus* A1 [58], *Streptomyces exfoliatus* CFS1068 [59], and *B. weihenstephaensis* PKD5 [60] had optimum incubation periods of four, six, and seven days, respectively.

Among the three *B. cytotoxicus* isolates selected, LT-1 produced the highest titles of proteolytic activity (17.1 ± 0.4 U/mL) in concordance with *Caldicoprobacter algeriensis*, a thermophilic bacteria isolated from hot spring, which produced 21.0 U/mL at 50 °C after 24 h of incubation [61].

Up to now, only five strains of *B. cytotoxicus* have been isolated around the world. The first one, *B. cytotoxicus* strain NVH 391-98T, was detected during a severe food poisoning outbreak in France in 1998, and the other four similar strains have been isolated since all are from food poisoning cases or food-related sources [37,62,63,64]. Nevertheless, the natural habitats of these strains have not been determined until now. Some authors have hypothesized that soil might be the natural habitat of *B. cytotoxicus*, considering the isolation of various strains from raw potatoes and various commercial potato products [38].

The members of the *B. cereus* group (or *B. cereus sensu lato*) are highly diverse bacteria, which can be found in varied environments on the Earth and have proved able to adapt to different habitats from cold to geothermal. Consequently, the growth temperature range of the strains in the *B. cereus* group is quite broad ranging from 4 to 50 °C [65]. Guinebretière et al. (2008) correlated the phylogenetic and phenotypic characteristics of the representative strains of the *B. cerus* group with their growth temperatures and thermal niches and demonstrated an ecotypic structure of the population, their classification in seven mayor phylogenetic groups. They showed conclusive evidence of how different members of this diverse group are adapted and diversified into novel environments by the modification of their growth temperature limits [66]. Particularly, the phylogenetic group VII, where *B. cytotoxicus* is included, is clearly associated with warm-temperature niches. However, it is probable that in diverse natural environments these moderately thermotolerant species might be overcome by more adapted ones, which could explain their low abundances and lack of retrieval in biodiversity studies. On the contrary, in dehydrated foods, the competition is out, and they might be favored by the selective effect of high temperatures used in the meal’s preparation processes [66]. The finding of particular thermal niches for particular species in the *B. cerus* group might help to comprehend the detection, for the first time, of *B. cytotoxicus* strains in a pristine natural habitat like the Domuyo geothermal area.

To the surprise of finding a rare species like *B. cytotoxicus* isolated from a natural pristine environment, we confirmed its identity by detecting the presence of the *cytK-1* gene. The *cytk* gene in the cytK-1 form is distinctive of the *B. cytotoxicus* NVH 391-98^T^ taxon, which, as already said, is the only thermotolerant taxon in the *B. cereus* group [37]. Due to its species sensitivity, the *cytK-1* gene has been exploited for a PCR diagnostic method to detect *B. cytotoxicus* strains and has been validated on numerous *B. cereus* group strains [34].

## 5. Conclusions

As we discussed, the original goal of our work was to assess the enzymatic hydrolytic potential, with special emphasis in keratinases, of extremophilic microbial species that inhabit the geothermal areas of Copahue and Dommuyo in the Neuquén Province in Argentina. The result of the enrichments and isolation under appropriate selective pressures was a collection of isolates (*B. cytotoxicus*) that were characterized by two molecular techniques. Our work reveals for the first time two novel characteristics of these species, i.e., its possible natural habit and its remarkably high proteolytic and keratinolytic capacities. The fact that *B. cytotoxicus* grow at moderate temperature and the isolates present the maximum proteolytic and keratinolytic activities at 50 °C may be a potential advantage for their use in several industries such as poultry and others that would benefit from the conversion of massive volumes of waste into profitable products.

## Figures and Tables

**Table 1:** Characteristics of the proteolytic *Bacillus* isolated from Domuyo. All the isolates developed proteolytic activity at 50 °C after 24 h, except for LT-6, LT-7, and LT-8 (in bold) that did it after 48 h.

Isolate	Colony Diameter (C; mm)	Hydrolysis Diameter (H; mm)	H/C Ratio	Taxonomic Identification	NCBI Accession Number
LT-1	5.09 ± 0.23	15.61 ± 0.26	3.06	*Bacillus cytotoxicus* (*B. cytotoxicus*)	KR559937
LT-2	4.33 ± 0.28	-		*B. licheniformis*	KY859160
**LT-6**	5.32 ± 0.28	10.21 ± 0.12	1.91	*B. licheniformis*	KR559939
**LT-7**	4.65 ± 0.33	9.56 ± 0.13	2.05	*B. licheniformis*	KR559940
**LT-8**	5.45 ± 0.28	10.13 ± 0.15	1.85	*B. licheniformis*	MF993534
Oll-13	8.34 ± 0.34	11.22 ± 0.20	1.34	*B. licheniformis*	KY859165
Oll-15	5.16 ± 0.11	14.15 ± 0.45	2.74	*B. cytotoxicus*	KR559942
Oll-16	6.14 ± 0.24	15.43 ± 0.21	2.51	*B. cytotoxicus*	KY559288
Oll-18	6.22 ± 0.36	14.32 ± 0.23	2.30	*B. cytotoxicus*	KR559943
Oll-30	6.18 ± 0.31	14.16 ± 0.13	2.30	*B. cytotoxicus*	KR559949
LT-32	7.22 ± 0.21	15.02 ± 0.12	2.10	*B. cytotoxicus*	KR559950
LT-34	6.33 ± 0.25	15.54 ± 0.54	2.45	*B. cytotoxicus*	KR559952
LT-35	7.17 ± 0.18	15.31 ± 0.12	2.13	*B. cytotoxicus*	KR559953

**Table 2:** Comparison of feathers degradation by aerobic, thermophilic bacteria.

	*B. cytotoxicus* LT-1	*B. licheniformis* Oll-15	*B. thermoruber* T1E [45]	*B. licheniformis* PWD-1 [41]	*S. thermonitrificans* MG104 [42]	*M. ruber* H328 [26]
Growth substrate	10 g·L intact chicken feathers	10 g·L intact chicken feathers	10 g·L intact goose feathers	10 g·L hammer-milled feathers	10 g·L intact chicken feathers	30 g·L intact chicken feathers
Incubation temperature	50 °C	50 °C	50 °C	50 °C	50 °C	55 °C
Incubation time	1 day	1 day	7 days	7–10 days	2–3 days	6 days

## Supplementary Materials

The following are available online at https://www.mdpi.com/2076-2607/8/6/796/s1, Table S1: Biochemical characterization of *B. cytotoxicus* strains isolated from the Domuyo geothermal area in the Neuquén Province, Argentina; Table S2: Enzymatic activity profiles of the isolates. Enzymatic activities were tested at 50 °C. + indicates the activity at 24 h (otherwise indicated), and - indicates no activity after 5 days. The last column indicates the accession number of each 16S rRNA gene sequence in the NCBI database.
