# Supplementary material for: Bacillus cytotoxicus Isolated from a Pristine Natural Geothermal Area Reveals High Keratinolytic Activity
Source: Microorganisms. 2020 May 26;8(6):796. doi: 10.3390/microorganisms8060796 (PMC7355561; doi:10.3390/microorganisms8060796)
Supplement: Supplementary file 1 [file microorganisms-08-00796-s001.pdf]

Supplementary Table 1. Biochemical characterization of *Bacillus cytotoxicus* strains isolated from Domuyo geothermal area in Neuquén province, Argentina.

Isolate	Catalase	Cytochrome Oxidase	Glucose	Lactose	H_2S production	Gas production	Nitrate reduction	Mannitol	Gelatin hidrolysis	Citrate	Methyl Red	Voges-Proskauer	Salt ($g.L^{-1}$)	Phylogenetic affiliation	NCBI Acc. Number
LT-1	+	+	+	+	-	-	+	-	+	-	+	-	25 - 100	*Bacillus cytotoxicus*	KR559937
Oll-15	+	+	+	+	-	-	+	-	+	-	+	-	25 - 100	*Bacillus cytotoxicus*	KR559942
Oll-16	+	+	+	+	-	-	+	-	+	-	+	-	25 - 100	*Bacillus cytotoxicus*	KY559288
Oll-18	+	+	+	-	-	-	+	-	+	-	+	-	25 - 100	*Bacillus cytotoxicus*	KR559943
Oll-30	+	+	+	+	-	-	+	-	+	-	+	-	25 - 100	*Bacillus cytotoxicus*	KR559949
LT-32	+	+	+	-	-	-	+	-	+	-	+	-	25 – 50	*Bacillus cytotoxicus*	KR559950
LT-34	+	+	+	-	-	-	+	-	+	-	+	-	25 - 50	*Bacillus cytotoxicus*	KR559952
LT-35	+	+	+	-	-	-	+	-	+	-	+	-	25 – 75	*Bacillus cytotoxicus*	KR559953

Supplementary Table 2. Enzymatic activity profile of the isolates. Enzymatic activities were tested at 50°C, + indicates activity at 24 hours (otherwise indicated), - indicates no activity after 5 days. The last column indicates the accession number of each 16S rRNA gene sequence in the NCBI Data base

**Isolate**	**Enzymatic activity**								**Phylogenetic affiliation**	**NCBI Acc. Number**
	keratinases	proteases	estearases	amylases	cellulases	inulinases	pectinases	xylanases		
LT-1	+	+	-	-	-	-	+	-	*Bacillus cytotoxicus*	KR559937
Oll-15	+	+	-	-	+	-	+	-	*Bacillus cytotoxicus*	KR559942
Oll-16	+	+	-	+	-	-	+	-	*Bacillus cytotoxicus*	KY559288
Oll-18	+	+	-	-	-	-	-	-	*Bacillus cytotoxicus*	KR559943
Oll-30	72 hs	+	-	-	-	-	-	-	*Bacillus cytotoxicus*	KR559949
LT-32	72 hs	+	-	-	-	-	-	-	*Bacillus cytotoxicus*	KR559950
LT-34	+	+	-	-	-	-	-	-	*Bacillus cytotoxicus*	KR559952
LT-35	+	+	-	-	-	-	-	-	*Bacillus cytotoxicus*	KR559953
